# Expertise, Working Memory and Articulatory Suppression Effect: Their Relation with Simultaneous Interpreting Performance

**DOI:** 10.5709/acp-0171-1

**Published:** 2015-06-30

**Authors:** Irene Injoque-Ricle, Juan Pablo Barreyro, Jesica Formoso, Virginia I. Jaichenco

**Affiliations:** 1 Psychology Research Institute, Faculty of Psychology, University of Buenos Aires (UBA); 2 National Scientific and Technical Research Council (CONICET)

**Keywords:** working memory, articulatory suppression, simultaneous interpreting

## Abstract

Simultaneous interpreting is a complex bilingual verbal activity that involves the auditory perception of an oral communication and the production of a coherent discourse. One of the cognitive functions underlying simultaneous interpreting is working memory. The aim of this work was to study the relationship between expertise, working memory capacity and articulatory suppression effect, and the ability to perform simultaneous interpreting. For this purpose, four working memory tasks and one simultaneous interpreting task were administered to thirty Spanish-speaking professional English interpreters. Results showed that simultaneous interpreting ability might be supported by the working memory´s capacity to store or process information, but also by the ability of the interpreter to cope with the articulatory suppression effect. We conclude that interpreters may have or develop resources to support the effect caused by articulatory suppression.

## Introduction

Simultaneous interpreting (SI) is a complex bilingual verbal activity that involves the auditory perception of an oral communication and the production of a coherent discourse. During SI, the verbal information is presented once, under conditions that restrict the processing time available and the amount of information that can be processed (Chernov, 2004). SI is an important tool in both academic and working contexts, because it allows people who speak different languages to communicate fluently and because an exact translation is essential to achieve good communication. SI is an interpretation modality that requires translating what a person is saying in a source language into a target language, and this is done during the production of speech. It is different from consecutive interpretation, where, to start translation, the interpreter waits for the speaker to finish. SI is highly complex because it involves listening, processing, and fluently translating speech with the smallest delay possible, all at the same time. In addition, the interpreter has to deal with the speaker’s verbal speed and the possible mistakes that she or he might make during the translation process.

SI involves different cognitive and linguistic competences. It is a task that requires efficient flexible attentional resources, language comprehension, and multiple phonological, phonetic, semantic and pragmatic subprocesses (Morelli, 2005). One of the cognitive functions underlying SI is working memory (WM), which is considered as one of the key factors of this interpretation process (Darò, 1989).

One of the currently prevailing models of WM is the embedded-processes model, proposed by Cowan (1988, 1999). It rests on the assumption that the processing performed by memory activation mechanisms, together with executive mechanisms and long term retrieval mechanisms result in an effective WM system. It is a limited memory system, regarding both time and capacity. Unless the representations are reactivated they fade within 10 to 20s, and it can hold 4±1 unrelated items (though the storage capacity can be increased by chunking). Nevertheless, there is a WM model that allows a better comprehension of the link between WM and SI: Baddeley´s multiple-component model (Baddeley, 1986, 2007, 2010; Baddeley & Hitch, 1974; Baddeley & Logie, 1999). Baddeley refers to WM as an active memory system responsible for the storage and simultaneous processing of information necessary to carry out complex cognitive activities, which allow the subject to comprehend and mentally represent the environment (Baddeley & Logie, 1999). According to Baddeley and Hitch’s (1974) original model (see also Baddeley, 1986, 2007, 2010; Baddeley & Logie, 1999), in the center of this memory system is the central executive, responsible for the control and regulation of two slave subsystems: the phonological loop, in charge of the temporary storage of verbal and acoustic information, and the visuospatial sketchpad which temporary holds visual and spatial information. According to Baddeley (1996), due to its impact on cognition the central executive is the most important component of WM. It is an attentional control system that is responsible for the activation of processes and representations that are held in long term memory. A classical measure used to assess the central executive is the Listening Span task (Daneman & Carpenter, 1980), which consists in the oral presentation of a series of sentences, and the participant’s evaluation of the semantic accuracy of each sentence as well as the serial recall of the last word of each sentence. The phonological loop maintains the verbal information temporarily stored active by using an articulatory rehearsal system, and this information can be used by the central executive or by the processes activated by it. The most frequently used measure of the phonological loop is the Digit Span task, where a series of digits is verbally presented, and the participants are requested to immediately serially recall the digits. The visual and the spatial information temporarily stored with the visuospatial sketchpad can also be used by the central executive or by the activated processes when necessary. The three components of WM have been related to a wide range of cognitive processes, both in childhood and adulthood. Some of these cognitive processes are language and vocabulary acquisition, reading comprehension, acquisition of arithmetic abilities, mental arithmetic, planning, learning of spatial routes, and intelligence (Alloway, Gathercole, Willis, & Adams, 2004; Anderson, Reder, & Lebiere, 1996; Anderson, Anderson, Northam, Jacobs, & Mikiewicz, 2002; Ashcraft, 1995; Burin, Duarte, Prieto, & Delgado, 2004; Carpenter, Just, & Shell, 1990; Carroll, 1993; Cowan, 2000; Dark & Benbow, 1990; Engle, Tuholski, Laughlin, & Conway, 1999; Garlick & Sejnowski, 2006; Gathercole & Baddeley, 1990; Injoque-Ricle, Calero, Alloway, & Burin, 2011; Kane & Engle, 2002; Liu, Schallert, & Carroll, 2004; Mizuno, 2005). In 2000 Baddeley included a new component in the WM model: the episodic buffer. This system serves as an interface between different systems, each one involving different sets of codes. This is possible due to the utilization of a common multi-dimensional code. It can be accessed by the central executive and can link together information held in the long term memory to form an episodic representation (Baddeley, 2000).

Within the long research tradition of the cognitive processes involved in SI, WM is one of the processes that have received most attention, both in theoretical writings and empirical studies. The three most accepted SI cognitive models postulate that WM has a crucial role in this type of interpretation (Darò & Fabbro, 1994; Gerver, 1975, 1976; Moser, 1978), although this role is generally limited to its storage aspect. One of the first cognitive models of SI is Gerver’s model (1975, 1976), which postulates a sequence of mental processes that occur during the interpretation process and includes a series of temporal storage systems for the different stages of text processing, one of which is WM. According to Gerver, the source text is held in an input buffer, and is processed in cooperation with long-term memory, which is in charge of activating the adequate linguistic units and is a strictly linguistic process. Once the information is processed, it goes to an output buffer where it can receive extra monitoring. Gerver also postulates that there are two separated buffers: one for the source language and another for the target language. Another process model is Moser’s model (1978), which gives WM a crucial role. In this model, WM is denominated generated abstract memory, and is a structural and functional component of the SI process. The generated abstract memory handles the storage of both semantically and syntactically processed text units, and, in cooperation with a conceptual base, the coding process. A third model is the one postulated by Darò and Fabbro (1994) that merges contemporary findings on SI and ideas about memory from cognitive psychology. This model is in line with the current memory system models (Timarová, 2008) and includes two memory systems: long-term memory and WM. WM is based on the original Baddelely and Hitch’s model (Baddeley, 1986; Baddeley & Hitch, 1974), and incorporates only the central executive and the phonological loop, but does not assign any role to the executive component. According to this model, WM is a passive storage of the source text, and the target text interferes with this storage function, limiting WM capacity.

On the one hand, the information processing performed by the central executive is rarely taken into consideration in the theoretical models or empirical studies (Timarová, 2008). On the other hand, there are few studies that relate SI performance and WM (Timarová, 2008). Most of the studies focus on the relationship between the expertise of simultaneous interpreters and WM. These studies generally use groups of trained interpreters, SI students, and people with a proficiency level of the target language. Some of these studies have found differences between the groups (e.g., Padilla, Bajo, Canas, & Padilla, 1995; Padilla, Bajo, & Macizo, 2005), but others have found no differences (e.g., Chincotta & Underwood, 1998; Liu et al., 2004).

Padilla et al. (1995) studied the need for interpreters to have a good WM. These authors proposed that an excellent WM is a pre-requisite for acquiring SI abilities, and that WM is improved with SI training. In order to test these hypotheses, they assessed both storage and processing capacities of WM in simultaneous interpreters and a control group (non-interpreters). For that purpose, they used *Digit Span* and *Listening Span*, two classic storage and processing WM measures. They found that simultaneous interpreters had better scores on both tasks than non-interpreters, concluding that they had better storage and processing capacities of WM. On the one hand, Christoffels, De Groot, and Waldorp (2003) found that SI performance is related to the performance on WM tasks, and concluded that WM span can underlie the ability to comprehend and produce simultaneously. On the other hand, Liu et al. (2004), administered *Listening Span* to SI beginner students, SI advanced students, and simultaneous interpreters with experience, and found no differences between the performance of each group on the WM tasks.

According to Christoffels and De Groot (2005), a source of difficulty on SI is that language comprehension and language production occur simultaneously. This can be explained by the articulatory suppression effect. Articulatory suppression implies that the articulation of irrelevant information during a verbal task affects the normal functioning of the phonological loop (Baddeley, 2007, 2010; Baddeley & Logie, 1999). According to Baddeley, this happens because this articulation prevents the subvocal rehearsal of the verbal input, and, on this case, the subvocal rehearsal of the source speech. Regarding this effect, Padilla et al. (1995) found no effect of articulatory suppression on expert interpreters and found a normal effect on interpreters with no experience and SI students, and postulated that experienced simultaneous interpreters are less affected by the demands of this effect. Padilla et al. (2005) replicated these results. They administered a *Word Span* task with and without articulatory suppression to professional interpreters, high WM span psychology students, and young professionals in language related students, and also found no articulatory suppression effect among the interpreters. The presence of the effect in the high WM span group allowed the authors to conclude that the absence of the articulatory suppression effect cannot be explained only by their larger WM capacity. Chincotta and Underwood (1998) also studied the effect of articulatory suppression on SI. They administered *Digit Span* with and without articulatory suppression to experienced simultaneous interpreters, SI students, and non-interpreter controls, and found no differences between the groups. Chincotta and Underwood postulated that because concurrent articulation of a verbal input during the production of verbal speech is common, expert simultaneous interpreters would not be affected by the articulatory suppression effect when compared with SI students and non-interpreter controls, but found no significant differences between the three groups in the performance on the task where irrelevant information had to be articulated.

Most studies have focused on whether WM capacity is different between simultaneous interpreters and non-interpreter controls, but only a few have focused on SI performance and its relationship to WM. Tzou, Eslami, Chen, and Vaid (2011) studied the effect of language proficiency and SI training on SI performance. They administered *Digit Span* and *Reading Span* to SI students and non-interpreter controls and found positive associations between WM measures and SI performance.

Based on the above, the aim of this work was to study i) the relationship between the experience of simultaneous interpreters and WM, with and without articulatory suppression, ii) the relationship between SI performance and WM, also with and without articulatory suppression, and iii) the predictive effect of the executive component of WM, the verbal storage component of WM, and expertise on SI performance of professional interpreters. We hypothesized that if an association was found between WM capacity and SI performance, WM training strategies could be designed to be included during the training process of simultaneous interpreters.

## Method

### Participants

Thirty Spanish-speaking professional English interpreters (26 females - 86.67%, and 4 males) with a mean age of 39.17 (*SD* = 7.81) voluntarily participated in the study. The interpreters had worked between 1 and 20 days per month (*X* = 6.95, *SD* = 5.18) (see Table 1 for descriptive statistics).

**Table 1.** Descriptive Statistics of Experiment

	Mean	*SD*	Min.	Max.	Skewness	Kurtosis
*SI performance*	22,27	6,02	6	30	-1,286	1,365
*Years of experience*	13,70	7,25	1	35	0,671	1,641
*Worked days per month*	6,95	5,18	1	20	0,431	-0,463
*Digit Span*	16,83	3,45	12	24	1,116	0,322
*Digit Span with AS*	12,17	5,42	1	24	0,414	0,385
*Listening Span*	7,67	2,23	4	13	0,669	0,093
*Listening Span with AS*	5,63	2,28	1	11	0,375	-0,117

*Note*. AS = Articulatory Suppression.

### Materials

#### Working Memory tasks

*Digit Span (Forward)* (ad hoc). This is a measure of the Phonological Loop according to Baddeley´s WM model, or verbal short term memory. Participants have to recall orally, in the correct order, digit sequences presented verbally. The number of digits to recall increases from two to nine, and three trials are presented for each number of digits. The task ends when two of the three trials with the same number of digits are incorrect.

*Listening Span* (ad hoc). This is a verbal measure of the Central Executive or verbal WM, originally created by Daneman and Carpenter (1980). Participants listen to a series of short sentences and have to decide whether they are true or false. Afterwards, they have to recall the last word of each sentence in the exact order in which they were presented. The number of sentences presented increases from two to six. Administration and stop criterion are the same as for the Digit Span task.

*Digit Span with Articulatory Suppression* (ad hoc). The aim of this task is to assess the effect of articulatory suppression on verbal short term memory. The procedure is the same as in the Digit Span task, but when listening to the digits, the participants have to say a word not related to the stimulus. The stimuli from this task are different from that of the Digit Span task.

*Listening Span with Articulatory Suppression* (ad hoc). This task allows assessing the effect of articulatory suppression on verbal WM. It is similar to the Listening Span task, but when listening to the sentences, the participants have to say a word not related to the stimulus. The sentences are different from those of the Listening Span task.

#### Simultaneous Interpreting task (ad hoc)

This is a task to assess SI ability. A pre-recorded 5-min. video conference on WM and dyslexia was presented. The interpreting was recorded and rated from 1 to 10 on three aspects: fluency, delay, and accuracy and quality. Afterwards the three scores were combined in a global SI performance score. Two independent bilingual raters graded each recording, with a high inter-rater reliability (*r* = .909, *p* < .001).

### Procedure

All interpreters were assessed in an individual session lasting approximately 30 min, which took place in a quiet office, free of distractions. The tasks were administered in the following sequence: *Digit Span*, *Digit Span with Articulatory Suppression*, *Listening Span*, *Listening Span with Articulatory Suppression*, and *Simultaneous Interpreting task*.

### Data analysis

A correlation analysis was conducted in order to assess the association between the variables of WM, expertise and SI performance. To study which WM component is a better predictor of SI performance, three multiple linear regression analyses were carried out. Finally a one-way analysis of variance (ANOVA) was conducted to compare high and low WM span interpreters.

## Results

Descriptive statistics for all the variables included in the study are shown in Table 1.

The correlation analyses showed positive significant correlations between SI and the WM variables and between SI and the number of days worked per month (see Table 2). The only variable that showed no significant correlation was *years of experience*. The association was stronger between the tasks including articulatory suppression.

**Table 2.** Pearson Correlations Among Simultaneous Interpreting (SI) Performance, Expertise Variables, and Working Memory Variables

	*Years of experience*	*Worked days per month*	*SI performance*
	*r*
*Years of experience*	-	-	-
*Worked days per month*	271	-	-
*SI performance*	.151	.500*	-
*Digit Span*	.207	.205	.428
*Digit Span with AS*	.349	.252	.543*
*Listening Span*	.454	.238	.410
*Listening Span with AS*	.226	.081	.540*

*Note*. * *p* < .05 using Bonferroni correction; AS = Articulatory Suppression.

A multiple linear regression model was tested (Model 1), including *SI performance* as dependent variable, and *days worked per month* and all four WM tasks as independent variables. The variable *years of experience* was not included in any of the models because it showed no significant correlation with the dependent variable. This initial model was statistically significant, *F*(5, 24) = 6.744, *p* < .001, R^2^ = .498, and the independent variables that predicted the dependent variable were *days worked per month* (β = .457; *t* = 3.288, *p* = .003) and *Listening Recall with Articulatory Suppression* (β = .581; *t* = 2.725, *p* = .02) (see Table 3).

**Table 3.** Multiple Linear Regression Model

		β	*T*	*p*	R^2^ corrected
Model 1	Constant		2,838	.009	.498
	*Worked days per month*	0,457	3,288	.003	
	*Digit Span*	0,118	0,705	.488	
	*Digit Span with AS*	0,377	1,550	.134	
	*Listening Span*	-0,519	-1,967	.061	
	*Listening Span with AS*	0,561	2,725	.012	

*Note*. Dependent variable: simultaneous interpreting performance; AS = Articulatory suppression.

A question that emerged from the regression’s results is which are the cognitive processes or strategies that allow simultaneous interpreters to cope with the articulatory suppression effect. To answer this question, we performed a new analysis, comparing high and low WM span interpreters, to determine if WM capacity could be one of the cognitive processes involved in this coping ability. The sample was divided into two groups. Interpreters with a WM span equal or higher than percentile 75 were included on the high WM span group (9 interpreters), and interpreters with a WM span equal or lower than percentile 25 were included on the low WM span group (8 interpreters). WM span was estimated from the performance on the *Listening Span* task, following the procedure carried out by Conway et al. (2005) and Engle (2001). Table 4 shows the frequency for each *Listening Span* score and the percentile values.

**Table 4.** Working Memory WM Span Frequency

	Frequency
3,00	9
3,50	4
4,00	9
4,50	5
5,50	1
6,00	2

*Note*. 25 percentile = 3.00; 50 percentile = 4.00; 75 percentile = 4.50

The one-way ANOVA compared the performance on the *Simultaneous Interpreting* task, the *Listening Span with Articulatory Suppression* task and the *Digit Span with Articulatory Suppression* task. Significant differences were found on all variables [*Simultaneous Interpreting*: *F*(1, 15) = 6.24, *MSE* = 7.79, *p* = .025; *Listening Span with Articulatory Suppression*: *F*(1, 15) = 21.90, *MSE* = 3.14, *p* < .01; *Digit Span with Articulatory Suppression*: *F*(1, 15) = 23.50, *MSE* = 18.27, *p* < .01], and on each task the high WM span group performed better than the low WM group (see Table 5).

**Table 5.** Descriptive Statistics for High and Low Working Memory WMspan groups

		Mean	*SD*
*SI performance*	Low Span	23,11	2,977
	High Span	26,50	2,563
*Listening Span with AS*	Low Span	4,22	1,716
	High Span	8,25	1,832
*Digit Span with AS*	Low Span	8,56	4,333
	High Span	18,63	4,207

*Note*. SI = simultaneous interpretation; AS = articulatory suppression.

In order to uncover some putative interaction effects, we performed an additional multivariate ANOVA (MANOVA) analysis, using as an independent variable, WM span, and as dependent variables *Simultaneous Interpreting*, *Listening Span with Articulatory Suppression*, and *Digit Span with Articulatory Suppression*. This analysis also showed that the high WM span group performed better on all tasks than the low WM span group, *F*(15, 72) = 2.47, *p* = .01.

## Discussion

Simultaneous Interpreting is a highly complex communicative activity that involves concurrent auditory perception of a source language and verbal expression in a target language (Chernov, 2004). SI is an important tool in both academic and working contexts. Because of this, it is important to understand the cognitive processes that restrict this ability. According to the most influential models of SI, one of its central cognitive processes is WM (Darò & Fabbro, 1994; Gerver, 1975, 1976; Moser, 1978). This memory system reflects active maintenance and simultaneously processing of information for relevant tasks. Baddeley (2007, 2010; see also Baddeley & Hitch, 1974) postulated a three-component model, with a Central Executive in charge of processing the information and regulating the slave subsystems: the Phonological Loop and the Visuospatial Sketchpad, two passive storage systems.

The first aim of this work was to study the relationship between SI experience and WM, with and without articulatory suppression. This analysis had the purpose to carry on the studies by Padilla et al. (1995), Chincotta and Underwood (1998), and Liu et al. (2004), who found opposite results regarding this relationship. Our results showed no significant association between both measures of SI experience and WM without articulatory suppression, in line with that found by Liu et al. (2004), and with articulatory suppression, in line with that found by Chincotta and Underwood (1998). This could implicate that experience in simultaneous interpreting has no effect on storage and processing capacity of WM.

Our second aim was to study the relationship between SI performance and WM with and without articulatory suppression. We found significant positive associations between SI performance and both measures of WM with articulatory suppression (the Phonological Loop with articulatory suppression and the measure of the Central Executive with articulatory suppression), but not between SI performance and the same measures without articulatory suppression. These results are not consistent with the results of the only other study in which this association was analyzed (Tzou et al., 2011). We conclude that our findings might indicate that interpreters have a good ability to support articulatory suppression, and that those with better ability to do so have a better SI performance. This result is in line with Elmer and colleagues’ findings (Elmer, Hänggi, Meyer, & Jäncke , 2011; Elmer, Meyer, Marrama, & Jäncke, 2011), which show that the linguistic training that results from SI modulates cerebral activities in the regions implicated in the top-down processing of auditory functions.

The results of the multiple linear regression analysis are in line with the results of the second correlation analysis. This regression analysis had the purpose to study the predictive role of two of the components of WM—Central Executive and Phonological Loop—and experience in SI performance. Since years of experience showed no significant association with SI performance, this variable was excluded from the analysis. The model tested was statistically significant and indicated that the variables that explained SI performance were interpreter’s experience (measured in days worked per month) and processing capacity of WM with articulatory suppression. This supports the previously presented idea that SI ability is supported not only by the capacity of WM to store or process information, but also by the ability of the interpreters to cope with the articulatory suppression effect. These interpreters may either have had or developed resources to support the effect caused by articulatory suppression, and these resources not only prevented the effect of articulatory suppression on the performance on SI tasks, but also potentiated the association between WM capacity and SI performance. The questions that arise from these findings are which is the cognitive process or strategy that allows simultaneous interpreters to cope with articulatory suppression and whether WM capacity enhances the performance of simultaneous interpreters.

The comparison of the interpretation performance of high and low WM capacity interpreters can give one possible answer to these questions. Results showed that high WM capacity interpreters performed better on the simultaneous interpreting task, which allows us to conclude that WM capacity is at least one of the abilities that underlie good simultaneous interpreting. Also, the performance on WM tasks with and without articulatory suppression also indicates that WM capacity might be underlying the ability to cope with this effect.

In future studies other variables, such as sustained and selective attention, or specific knowledge of the material being interpreted, should be included to determine their relation to the ability to interpret simultaneously and to cope with the articulatory suppression, and also to estimate the specific weight of WM capacity on the ability to simultaneously interpret well and to cope with articulatory suppression effect.

## Author’s Note

This research was supported by the Consejo Nacional de Investigaciones Científicas y Técnicas (Res. Nş 329/10) and by the Secretaría de Ciencia y Técnica, Universidad de Buenos Aires (UBACyT P052, Directors: Dr. N. Leibovich de Figueroa & Dr. V. Schmidt).
